# Repaying Student Loan Debt: Implications for Older Women’s Financial Well-Being and Retirement Planning

**DOI:** 10.1093/geroni/igaa057.2434

**Published:** 2020-12-16

**Authors:** Julie Miller, Samantha Brady, Alexa Balmuth, Lisa D’Ambrosio, Joseph Coughlin

**Affiliations:** 1 MIT AgeLab, Cambridge, Massachusetts, United States; 2 MIT, Cambridge, Massachusetts, United States

## Abstract

Previous research demonstrates that student loan debt disproportionately affects women and is being repaid increasingly by older adults. Utilizing data collected through a mixed methods study at the MIT AgeLab (involving focus groups and a large national survey), we examine the impacts of repaying student loans on older women’s financial wellbeing. Findings focus on multiple areas, including the timing in which older women repaying student loans were able to pay down other debts, change jobs, get married, and pursue further education. Analysis also revealed that student loans impacted the amount that 63% of older women in the study were able to contribute to their retirement savings. Findings from this study suggest that repaying student loan debt for oneself and/or a family member can compromise older women’s near-term and longer-term financial being. Policy reforms are needed to support older women repaying student loans.

